# Serrated Chips Formation in Micro Orthogonal Cutting of Ti6Al4V Alloys with Equiaxial and Martensitic Microstructures

**DOI:** 10.3390/mi10030197

**Published:** 2019-03-20

**Authors:** ZeJia Zhao, Suet To, ZhuoXuan Zhuang

**Affiliations:** State Key Laboratory of Ultra-Precision Machining Technology, Department of Industrial and Systems Engineering, The Hong Kong Polytechnic University, Hung Hom, Kowloon, Hong Kong SAR, China; ze-jia.zhao@connect.polyu.hk (Z.J.Z.); damon.zhuang@connect.polyu.hk (Z.X.Z.)

**Keywords:** serrated chips, Ti6Al4V alloy, micro orthogonal machining, martensite, cutting and thrust forces

## Abstract

The formation of serrated chips is an important feature during machining of difficult-to-cut materials, such as titanium alloy, nickel based alloy, and some steels. In this study, Ti6Al4V alloys with equiaxial and acicular martensitic microstructures were adopted to analyze the effects of material structures on the formation of serrated chips in straight line micro orthogonal machining. The martensitic alloy was obtained using highly efficient electropulsing treatment (EPT) followed by water quenching. The results showed that serrated chips could be formed on both Ti6Al4V alloys, however the chip features varied with material microstructures. The number of chip segments per unit length of the alloy with martensite was more than that of the equiaxial alloy due to poor ductility. Besides, the average cutting and thrust forces were about 8.41 and 4.53 N, respectively, for the equiaxed Ti6Al4V alloys, which were consistently lower than those with a martensitic structure. The high cutting force of martensitic alloy is because of the large yield stress required to overcome plastic deformation, and this force is also significantly affected by the orientations of the martensite. Power spectral density (PSD) analyses indicated that the characteristic frequency of cutting force variation of the equiaxed alloy ranged from 100 to 200 Hz, while it ranged from 200 to 400 Hz for workpieces with martensites, which was supposedly due to the formation of serrated chips during the machining process.

## 1. Introduction

To date, Ti6Al4V alloy is increasingly applied within the aerospace field due to its low weight, superior strength, and corrosion resistance [1,2]. Generally, the mechanical properties of the Ti6Al4V alloy are determined by its material microstructures, mainly including phase composition and grain size. Equiaxial and martensitic microstructures are two important types of structures for Ti6Al4V alloy [3]. An alloy with martensite can be obtained through high-temperature annealing (above β phase transformation temperature) followed by water quenching. The two-phase alloy with equiaxial grains usually has a balance of strength, ductility, and corrosion resistance, while alloy with martensite generally possesses high strength and corrosion resistance but low ductility [4].

Ti6Al4V alloy is regarded as one of the difficult-to-machine materials due to the low thermal conductivity and elastic modulus in traditional and ultraprecision machining [5,6,7]. Serrated chip (or saw tooth chip) formation is an important feature during the machining of Ti6Al4V alloys. The generation of serrated chips is totally different from the formation of continuous chips that are usually obtained in cutting most easy-to-machine materials, such as copper, aluminum, and most of their alloys [8,9,10]. The chips can take away the majority of generated heat because of the superior thermal conductivity of easy-to-cut materials. However, heat is not prone to being dispersed during the machining hard-to-cut alloys because of their poor thermal conductivity or fracture properties, resulting in serrated chip formation according to the thermal softening effect or periodical crack generation [11,12,13]. 

Two theories, that is, adiabatic shear band theory and periodical cracks formation theory, are the most widely accepted mechanisms for analyzing the generation process of serrated chips. Each theory works in a certain condition, and no consensus has been achieved on serrated chip formation in the machining of Ti6Al4V alloys. Komanduri and Von Turkovich [14] proposed that the generation of an adiabatic shear band gives rise to the formation of serrated chips in the high-speed machining of titanium alloys. If thermal softening predominates over strain hardening, shear localization can occur in the primary cutting zone. This contributes to a reduction of the required stress to overcome plastic deformation of a workpiece [15]. Sun et al. [16] proposed that severe plastic deformation in the narrow shear band results in the formation of serrated chips, and also found the formation of serrated chips could induce a periodical cutting force evolution. The periodical crack initiation and propagation between two segments of one serrated chip is another theory to investigate the chip-forming mechanism. Vysa and Shaw [17] reported the detailed crack generation process in the orthogonal cutting of difficult-to-cut AISI 1045 steel. Jiang and Rajiv [18] discussed that the serrated chip morphology was primarily caused by crack initiation and propagation during the machining of Ti6Al4V alloy. In addition, the formation of serrated chips was successfully simulated by using various models, including the Johnson–Cook (JC) law [19,20], modified JC law [21,22] and tangent hyperbolic (TANH) model [23]. Besides, the JC damage law and ductile fracture criterion [24,25] were adopted as criteria to analyze the fractural characteristic in discussing the saw tooth chip formation of titanium and its alloys.

Material microstructures also significantly affect the machinability of a workpiece during the machining process. Nouari and Makich [26,27] reported that tool wear was greatly influenced by the microstructures of titanium alloys. Ahmadi et al. [28] proposed that small grain size and low content of β phase could result in high cutting forces in the micro milling of Ti6Al4V alloys. Though some research has been carried out on studying the effects of microstructures on tool wear and cutting forces in the traditional machining of Ti6Al4V alloys, little research has been conducted on comparing the chip features from the perspective of material microstructures of the Ti6Al4V alloys in micro cutting. As strength and ductility vary between microstructures, the machinability of the Ti6Al4V alloys should also show different characteristics that can be indirectly reflected from the features of cutting chips. Hence, this research aims to investigate the effects of equiaxial and martensitic microstructures on the serrated chip formation of Ti6Al4V alloys via micro orthogonal machining.

## 2. Experimental Procedures

**Figure 1:**
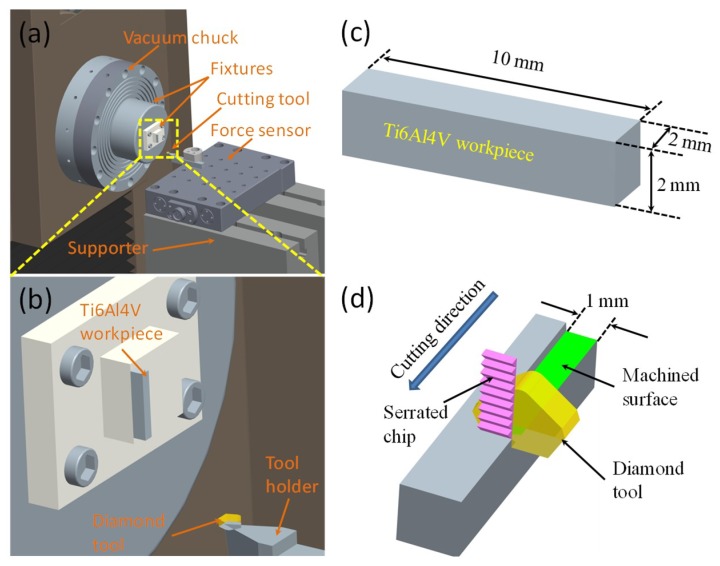
(**a**) Schematic set up of the micro orthogonal cutting and (**b**) the corresponding magnified image; (**c**) dimensions of the workpiece, and (**d**) serrated chip formation in cutting.

Ti6Al4V alloy with a diameter of 3 mm was utilized as the raw material. The alloy was first cut into six pieces with a length of about 10 mm and half of these were then treated for 2 min using an electropulsing device (THDMIII). The voltage and frequency of the electropulsing were 50 V and 400 Hz, respectively. After the electropulsing treatment (EPT), samples were quenched immediately in water (25 °C). Subsequently, all EPT-treated and original samples were cut into rectangular shapes using wire electrical discharge machining (WEDM) to then carry out micro orthogonal machining. The dimensions of the workpiece are shown in Figure 1c.

An ultraprecision machine (Moore Nanotech 350FG, Nanotechnology Inc, Swanzey, NH, USA) was used for the micro orthogonal cutting of the Ti6Al4V workpiece and the setup is schematically shown in Figure 1a,b. The head width, rake angle, and front clearance angle of the fresh flat diamond tool were 2 mm, 0°, and 10°, respectively. Rough machining with another diamond tool (nose radius of 1.103 mm, rake angle of 0°, and front clearance angle of 12.5°) was first carried out on the workpiece to obtain a flat surface on the ultraprecision machine. Then, micro orthogonal machining with a cutting width of 1 mm, cutting speed of 60 mm/min, and cutting depth of 3 µm was carried out on the workpiece, and machining was stopped at the cutting length of 6 mm. Figure 1d briefly illustrates the details of the micro orthogonal machining process. A Kistler force sensor (9256C1, Kistler Corporation, Winterthur, Switzerland) was installed under the cutting tool to measure variation in cutting and thrust forces. The measured frequency was set as 100 kHz, so frequencies lower than 50 kHz could be accurately calculated in the power spectrum density analysis by consideration of the Nyquist limit. An optical microscope (LEICA DFC 450, Leica, Wizlar, Germany) was utilized for metallographic observation. The electronic microstructures of the workpiece and cutting chips were measured by a Tescan VEGA3 scanning electron microscope (SEM, Tescan, Brno, Czech Republic). The compressive stress–strain test was conducted on a MTS 810 material testing system (MTS systems corporation, Eden Prairie, MN, USA) with a strain rate of 0.01 s^−1^, and the nano-hardness was measured using a Nano Indenter G200 (Keysight Technologies, Wokingham, UK).

## 3. Results and Discussion

### 3.1. Microstructures, Stress–Strain Curves, and Nano-Hardness

**Figure 2:**
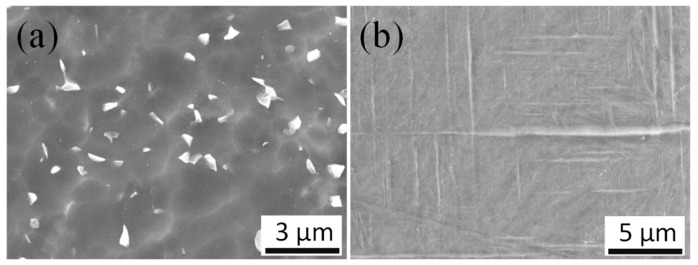
SEM microstructures of the (**a**) original alloy, and (**b**) electropulsing treatment (EPT)-treated Ti6Al4V alloy.

Figure 2 shows the material microstructure of the original and EPT-treated Ti6Al4V workpieces. The microstructure of the original alloy consisted of equiaxial β grains (bright white particles) and α phase, and the average size of the grains was about 600 nm. The EPT treated alloys were composed of orthorhombic acicular martensite α and the width of the martensite ranged between 100 and 500 nm.

**Figure 3:**
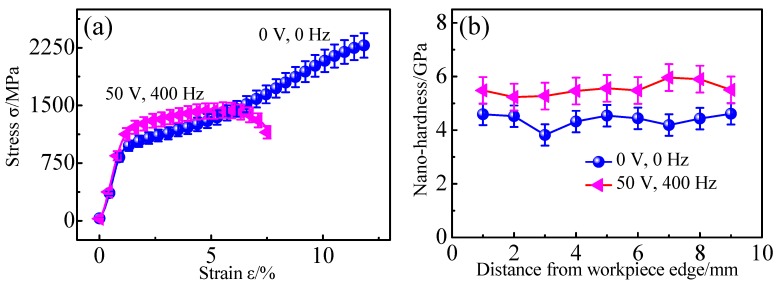
(**a**) Stress–strain curves, and (**b**) nano-hardness of the alloys without EPT treatment and with EPT of 50 V, 400 Hz.

The stress–strain curves and nano-hardness of the alloy with equiaxial β grains and martensitic structures are shown in Figure 3. It can be clearly seen from Figure 3a that the yield stress of the EPT treated alloy was higher than that of the original alloy, reaching about 1400 MPa. However, the ductility of the alloy after electropulsing was much lower in comparison with the original. As the ultimate strength of the treated alloy was also smaller than the alloy without treatment, the stress required to fracture should be less for the treated alloy. The nano-hardness of the workpiece is shown in Figure 3b. The average nano-hardness of the treated alloy increased by 26.3% compared with the original alloy with an average nano-hardness of about 4.384 GPa. The increase in nano-hardness was primarily due to the increase of the yield stress, which required high stress to overcome plastic deformation.

### 3.2. Cutting Chips

**Figure 4:**
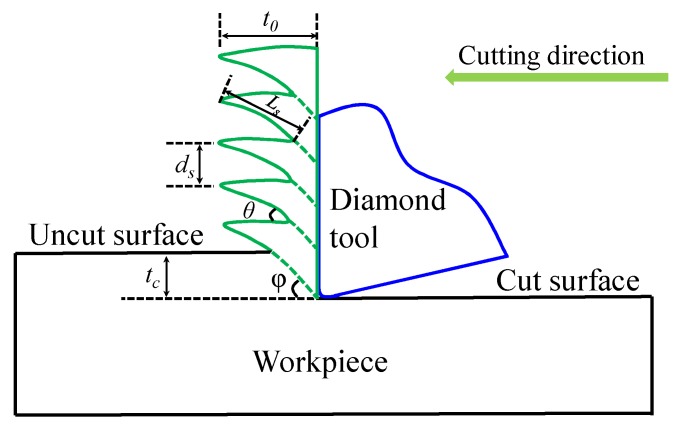
A schematic diagram of the serrated chip formation process.

Figure 4 schematically illustrates the serrated chip formation process in the micro orthogonal machining. Chip thickness *t_0_*, distance of chip segment *d_s_*, serrated chip angle *θ*, and segment length *L_s_* are the main features of one continuous serrated chip.

**Figure 5:**
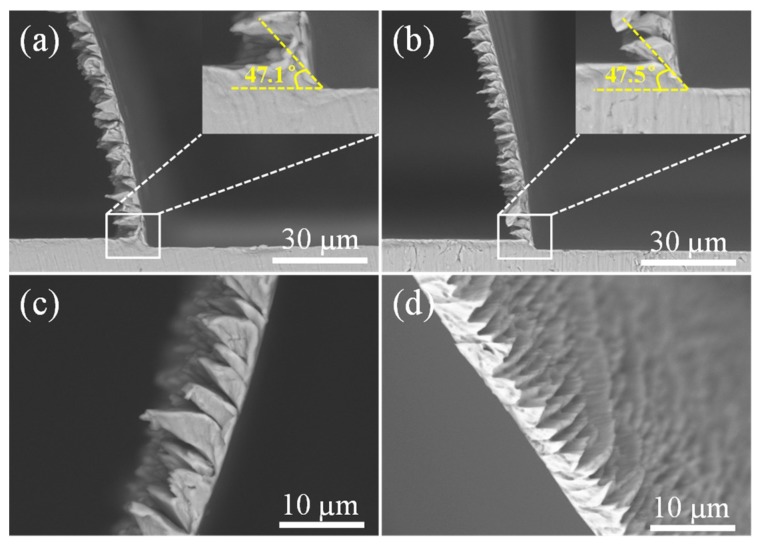
Serrated chip morphologies for (**a**,**c**) original alloys and (**b**,**d**) EPT-treated alloys.

Figure 5 compares the chip morphologies of the alloys after micro orthogonal cutting. Continuous serrated chips consisting of a large number of consecutive chip segments were formed at an extremely low cutting speed (60 mm/min), which is different from a previous study that proposed that continuous serrated chips could be observed at cutting speeds higher than 75 m/min in the traditional machining of Ti6Al4V alloys [16]. This may be because the cutting depth in ultraprecision micro cutting is much smaller in comparison to traditional machining, which usually has a cutting depth of more than several hundred micrometers.

Shear angle is the angle between the shear direction in the primary deformation zone and cutting speed direction, which is an important parameter in evaluating material removal and cutting chip thickness [12,29]. In Figure 5a,b, the measured shear angles were almost equal for the alloy with equiaxial and martensitic microstructures, reaching values of 47.1° and 47.5°, respectively. This means that microstructures had slight effects on the shear angle in micro orthogonal cutting, which was also proposed by Sun et al. [16]. However, the number of chip segments per unit length varied with the microstructure. The continuous serrated chips for the EPT-treated alloy with the martensitic microstructure had more segments per unit length in comparison to the alloy without treatment, as compared in Figure 5c,d. Furthermore, the length of one chip segment of the martensitic alloy was shorter than the original and the serrated chip angle also varied between microstructures.

As microcracks throughout two neighboring chip segments were obviously found for both alloys, the serrated cutting chips were supposedly a result of periodical crack initiation and propagation between two adjacent segments during the micro orthogonal machining of Ti6Al4V alloys. Chip formation was affected by the cutting conditions, cutting tools, and materials [30,31,32]. As machining conditions and cutting tools were all the same during micro orthogonal cutting, the different serrated chip features can be attributed to the various material microstructures. The ductility of the alloy with acicular martensite was much lower than the alloy with equiaxial microstructures, according to the stress–strain curve shown in Figure 3. Therefore, the martensitic alloy showed a more “brittle” feature than the equiaxial alloy. Hence, fracture failure is prone to occur for an alloy with a martensitic microstructure, which contributes to more chip segments per unit length.

### 3.3. Cutting and Thrust Forces

**Figure 6:**
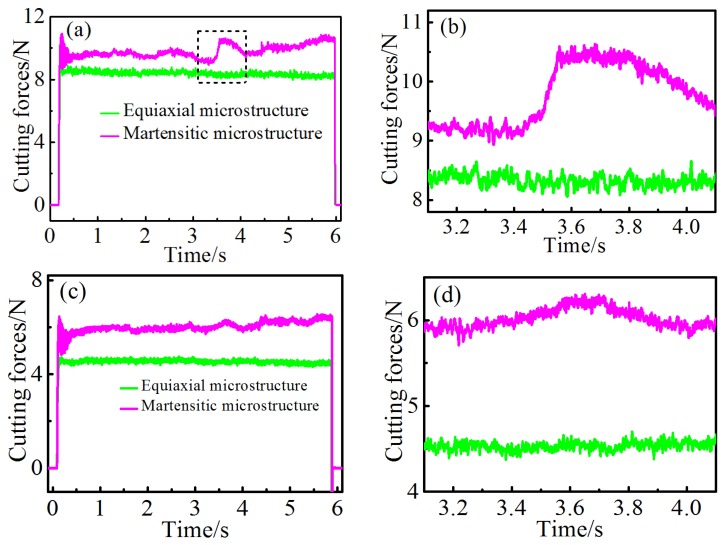
(**a**) Cutting force variation, and (**b**) corresponding magnified image; (**c**) thrust force variation, and (**d**) corresponding magnified image.

Figure 6 compares the cutting and thrust forces variation for the two types of alloy with respect to microstructures. The average cutting and thrust forces were about 8.41 N and 4.53 N, respectively, for all the equiaxial two-phase alloys, which was consistently lower than in those with martensitic structure. As the yield stress of plastic deformation of the alloy with martensite was higher than the original, the cutting force required to overcome flow stress should also be larger for the martensitic alloy.

**Figure 7:**
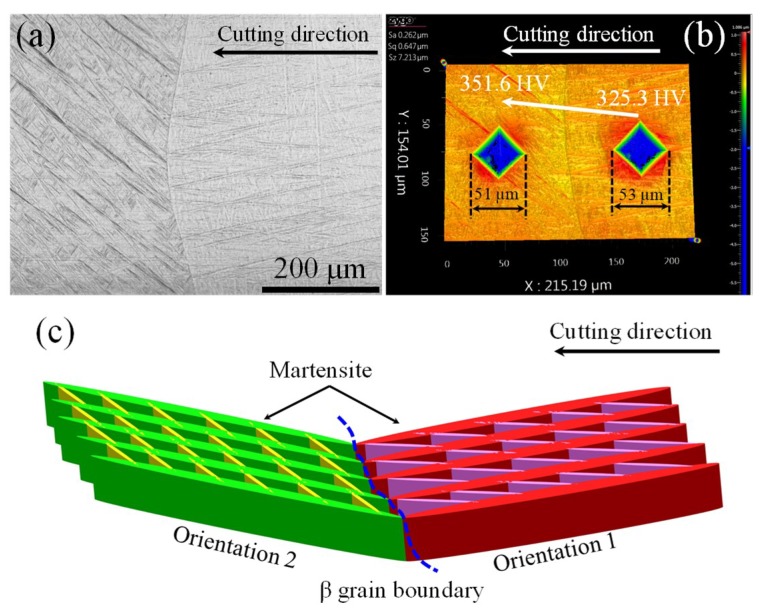
(**a**) Microstructures of the martensitic Ti6Al4V alloy at the region with force increase, (**b**) comparison of indentation markers and Vickers hardness, and (**c**) a sketch of martensitic orientation change.

Furthermore, the evolution of cutting and thrust forces of the equiaxial alloy showed a stable trend during the whole machining process, while obvious force fluctuations could be clearly seen in the cutting and thrust force curves. As the martensitic distribution of the three workpieces was different, the forces also showed various variation trends. Figure 6a,c illustrate the cutting and thrust force of one workpiece with microstructures shown in Figure 7. Obvious force fluctuations were observed in the curves, especially in the region marked by the black dash rectangle in Figure 6a, as the average cutting force showed a sudden increase from 9.18 to 10.45 N. A similar thrust force, increasing from 5.92 to 6.23 N, was also found during machining, however the increase was not obvious when compared with the cutting force.

The cutting and thrust force variation was primarily due to the effects of different martensitic orientations, as shown Figure 7. The microstructure of the region where the cutting and thrust force experienced a sudden increase is shown in Figure 7a. Initial β grain boundary was observed between two orientations of martensite and the martensitic density of the left region was slightly more intensive than that of the right region. Figure 7b compares the two indentation marks of the Vickers hardness and shows that measured hardness in the left region was slightly higher than the right region, which indicates that the required stress for plastic deformation was larger than the right region.

The martensite density in left region was more intensive than that in the right region, which increased the number of phase boundaries. Hence, the required flow stress for plastic deformation should also be higher in the left region, which agrees with previous studies showing that thin and small martensites usually give rise to high yield and ultimate strength [3,33].

In addition, the primary martensitic orientation of the two sites was totally different at the two sites, which is schematically shown by different colors in Figure 7c. The equiaxial Ti6Al4V alloy was composed of hexagonal closest packing (HCP) α phase and body centered cubic (BCC) β phase at room temperature. However, the crystallographic structure of the Ti6Al4V alloy with acicular martensite mainly consisted of HCP α phase, and only 0.4% of BCC β phase was obtained based on the previous study [34]. Hence, plastic deformation of the alloy was significantly affected by the martensitic α phase instead of β phase.

**Figure 8:**
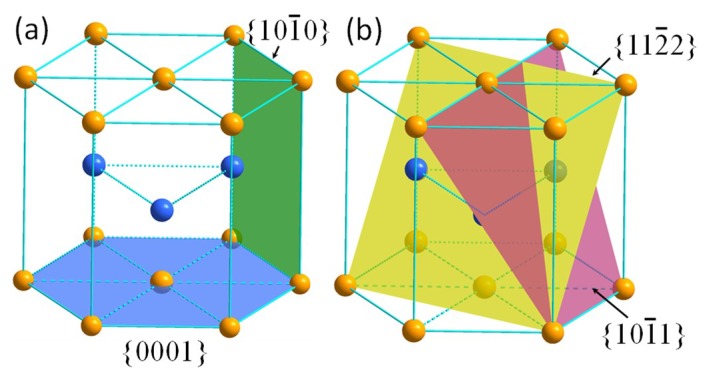
Schematics of crystalline structure of hexagonal closest packing (HCP) titanium: (**a**) $\left\{10\bar{1}0\right\}$ and {0001} planes; (**b**) $\left\{10\bar{1}1\right\}$ and $\left\{11\bar{2}2\right\}$ planes.

For deforming HCP titanium, slipping modes mainly included prismatic slip of $\left\{10\bar{1}0\right\}$ plane, basal slip of {0001} plane, and pyramidal slip of $\left\{10\bar{1}1\right\}$ plane. The slipping direction of the three systems was all in the direction of $<11\bar{2}0>$. The first-order and second-order pyramidal <c+a> slips with direction of $<11\bar{2}3>$ on planes of $\left\{10\bar{1}1\right\}$ and $\left\{11\bar{2}2\right\}$ were other deformation modes for HCP titanium. The prismatic and basal slipping planes are represented by the green and blue color in Figure 8a, respectively, and the pyramidal slipping planes of $\left\{10\bar{1}1\right\}$ and $\left\{11\bar{2}2\right\}$ are shown by the plum and yellow colors in Figure 8b, respectively. In addition, some twinning systems, such as $\left\{10\bar{1}2\right\}<10\bar{1}1>$, $\left\{11\bar{2}1\right\}<\bar{1}\bar{1}26>$, and $\left\{10\bar{1}1\right\}<\bar{1}012>$, could also be activated to assist deformation.

The prismatic <a> slip of $\left\{10\bar{1}0\right\}<11\bar{2}0>$ was the most prone to be activated in comparison to other systems during deformation HCP titanium due to the relatively low critical resolved shear stress (CRSS). However, it was not the only slip model because five independent slip systems are required to accommodate the plastic deformation, therefore the basal <a> slip, pyramidal <a> slip, pyramidal <c+a> slip, and twinning might also be activated during deformation to fulfil this requirement. As the primary martensitic orientation was different at the left and right sides of the β grain boundary, the activation of deformation modes should also vary with the orientations, which is similar to the research conducted by To et al. [34] into the ultraprecision rotary cutting of Ti6Al4V alloy with martensitic microstructure. Hence, the cutting force increase induced by the change of martensite orientations indicates that some deformation models with high CRSS were activated when the diamond tool passed through the β grain boundary.

### 3.4. Power Spectral Density (PSD) of Cutting Forces

**Figure 9:**
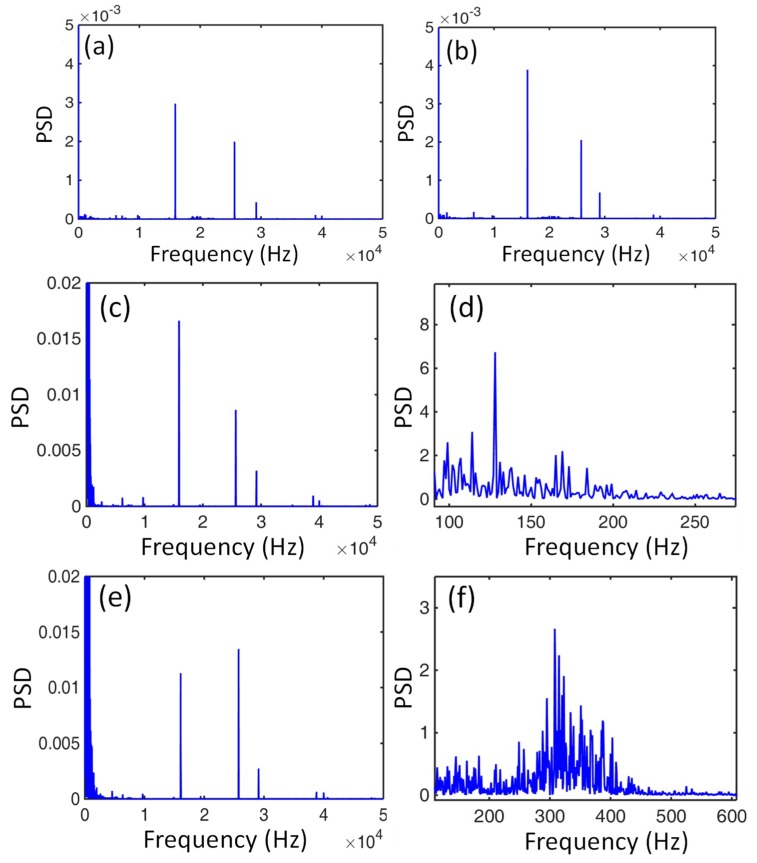
Power spectral density (PSD) analyses for air cutting of the alloy with (**a**) equiaxed and (**b**) martensitic microstructure; (**c**–**f**) PSD analyses under different frequencies for the equiaxed and martensitic microstructures, respectively.

PSD analysis via transforming time domain to frequency domain by the fast Fourier transform (FFT) was adopted to investigate the cutting force signals of the Ti6Al4V alloys with two types of microstructure, as shown in Figure 9. Three high frequencies at about 16.05, 25.75, and 29.10 kHz could be clearly observed in the air cutting conditions when the diamond tool did not make contact with either the original or EPT-treated alloy, and the corresponding PSD values were fairly small, as shown in Figure 9a,b. Hence, the three high frequencies should be intrinsic background frequencies generated by the machining system.

During the machining process when the diamond tool was consistently cutting the workpiece, the characteristic frequency of the cutting force signals was mainly in a low frequency scale with a large PSD value in comparison to the three high intrinsic background frequencies for both of the workpieces, as shown in Figure 9c–f. The characteristic frequency of cutting force variation of the equiaxial two phase alloy ranged from 100 to 200 Hz, while it ranged from 200 to 400 Hz for the workpiece with martensites.

The cutting force variation was estimated from the serrated chip feature in machining Ti6Al4V alloys according to Sun et al. [16]. The frequency *f* of cutting force variation could be calculated by the segment length and cutting speed (*f* = *v/ L_s_*). Since segment lengths measured from Figure 5 ranged from 4.34 to 8.26 µm and 2.17 to 3.91 µm, the calculated vibration frequencies were in ranges of 121.07–230.41 Hz and 255.75–460.83 Hz for the original and EPT-treated alloy, respectively.

## 4. Conclusions

The serrated chip formation of Ti6Al4V alloys was experimentally investigated in this study, which provides useful instructions for analyzing chip features with respect to equiaxed and martensitic microstructures during the micro machining of Ti6Al4V alloys. Conclusions could be drawn as follows:

(1) The needlelike martensitic microstructure of the Ti6Al4V alloy with widths ranging from 100 to 500 nm could be obtained by electropulsing treatment for 2 min followed by water quenching.

(2) The shear angle was slightly affected by the microstructures of the Ti6Al4V alloy. However, the chip features varied significantly between alloy microstructures. The number of chip segments per unit length of the martensitic alloy were more than the original alloy due to poor ductility, however the segment length of the former was shorter than the latter.

(3) The average cutting and thrust forces remained stable at about 8.41 and 4.53 N for the equiaxial Ti6Al4V alloy, which were both lower than those with martensitic microstructures that demonstrated fluctuating cutting force variation. Besides, the sudden increase of cutting force from 9.18 to 10.45 N for the martensitic Ti6Al4V alloy was due to the change of martensitic orientations.

(4) The characteristic frequency of cutting force signals for the equiaxed alloy ranged from 100 to 200 Hz, while it ranged from 200 to 400 Hz for the alloy with martensites, according to the power spectral density (PSD) analyses. The varying frequencies were supposedly due to the different serrated chip features in the machining process.
